# Age-adjusted Charlson Comorbidity Index (ACCI) is a significant factor for predicting survival after radical gastrectomy in patients with gastric cancer

**DOI:** 10.1186/s12893-019-0513-9

**Published:** 2019-05-27

**Authors:** Jian-Xian Lin, Ying-Qi Huang, Jian-Wei Xie, Jia-bin Wang, Jun Lu, Qi-Yue Chen, Long-long Cao, Mi Lin, Ru-Hong Tu, Ze-Ning Huang, Ju-Li Lin, Ping Li, Chang-Ming Huang, Chao-Hui Zheng

**Affiliations:** 1 Department of Gastric Surgery, Fujian Medical University Union Hospital, Fuzhou, Fujian Province China; 2 Department of General Surgery, Fujian Medical University Union Hospital, Fuzhou, Fujian Province China; 3 Key Laboratory of Ministry of Education of Gastrointestinal Cancer, Fujian Medical University, Fuzhou, Fujian Province China

**Keywords:** Gastric adenocarcinoma, ACCI, Gastrectomy, Outcomes

## Abstract

**Introduction:**

To assess the ability of the Age-Adjusted Charlson Comorbidity Index (ACCI) to predict survival after radical gastrectomy in patients with gastric cancer (GC).

**Method:**

Data from patients with GC who underwent radical gastrectomy from January 2008 to December 2012 in Fujian Medical University Union Hospital were retrospectively analyzed. Patients were categorized into either high ACCI group or low ACCI group based on the effect of ACCI on long-term GC prognosis. 1:1 propensity score matching (PSM) was used to reduce confounding bias. To further analyze the impact of ACCI on the long-term prognosis of patients after radical gastrectomy, a nomogram was built based on the Cox proportional hazards regression model.

**Results:**

A total of 1476 patients were included in the analysis. After PSM, there was no statistically significant differences in tumor location, tumor size and tumor stage between low ACCI group (429 cases) and high ACCI group (429 cases) (all *P* > 0.05). Before and after PSM, the incidence of postoperative complications in high ACCI group was significantly higher than that in low ACCI group (*P* < 0.05). The 5-year overall survival rate (OS) in low ACCI group was significantly higher than that in high ACCI group. Multivariate analysis showed that ACCI was an independent risk factor for OS (*P* < 0.05). The Harrell’s C-statistics (C-index) of TNMA, a prognostic evaluation system combining ACCI and TNM staging system, was significantly higher than that of TNM staging system in both the modeling and validation groups (all *P* < 0.05).

**Conclusions:**

ACCI was an independent risk factor for the long-term prognosis of GC patients after radical gastrectomy that could effectively improve the predictive efficacy of the TNM staging system for GC.

**Electronic supplementary material:**

The online version of this article (10.1186/s12893-019-0513-9) contains supplementary material, which is available to authorized users.

## Background

Although the incidence and mortality of GC showed a decreasing trend globally, [1], GC remains the fifth most common malignant tumor worldwide and the third leading cause of death (723,000 people die from GC, accounting for 8.8% of the world’s population) [2]. With the increase of population age and life expectancy, the proportion of elderly patients with gastric cancer also increases. Some studies showed that people over 70 years old account for more than 30% of patients with GC [3]. However, as the functional reserve of the human body tends to decline with age, elderly patients often have more comorbidities [4] and are more likely to have postoperative complications including death [5–9].

In 1994, Charlson et al. established a new scoring system, ACCI, to predict the incidence of complications during the perioperative period [10]. Recently, investigators have determined the predictive value of the ACCI on the long-term prognosis of patients with other malignant tumors (such as ovarian cancer, prostate cancer, pancreatic cancer, and colorectal cancer) [11–15]. Although the effect of the ACCI on the short-term prognosis of patients with GC has been reported in previous studies [16, 17], its predictive value on the long-term prognosis of patients with GC has not been reported. Therefore, the clinicopathological data of patients with radical gastrectomy from 2008 to 2012 were included in our study to evaluate the predictive value of the ACCI on the long-term prognosis of patients with GC.

## Methods

### Database

Data from patients with GC who underwent gastrectomy in the department of gastric surgery of the Affiliated Union Hospital of Fujian Medical University from January 2008 to December 2012 were collected and retrospectively analyzed. Patients who met the following criteria were included in this study: (1) pathologically diagnosed with gastric carcinoma before gastric surgery; (2) preoperative imaging examination (e.g., chest radiography, ultrasound examination, abdominal computed tomography) showed that the tumor had not invaded the surrounding organs, and there was no evidence of metastasis; and (3) patient underwent radical gastrectomy and proved to be an R0 excision after operation. Patients who had a previous history of gastric surgery or underwent neoadjuvant chemotherapy, or with gastric neuroendocrine tumor confirmed postoperatively were excluded. Thus, a total of 1476 patients were enrolled. The extend of gastrectomy and dissection of lymph nodes were performed according to the fourth edition of Japanese guidelines for the treatment of gastric cancer [18]. Tumor staging was based on the eighth cancer staging manual of American Joint Committee on Cancer (AJCC) [19].

### The definition of ACCI

The ACCI, as defined by Charlson et al., is a combination of the age equivalence index and Charlson Comorbidity Index (CCI) [12]. For patients who over 40 years old, the cumulative score was 1 point for each additional 10 years of age (e.g.,1 point for those aged 50–59 years, 2 points for those aged 60–69 years, and so on), and the score for age is added to the CCI (e.g., 0,1,2,3) (Table 1). A completely resolved condition (i.e., a history of pneumonia) or a history of current inactive surgery (i.e., a history of cholecystectomy) is not considered as a comorbid disease [20].

**Table 1:**
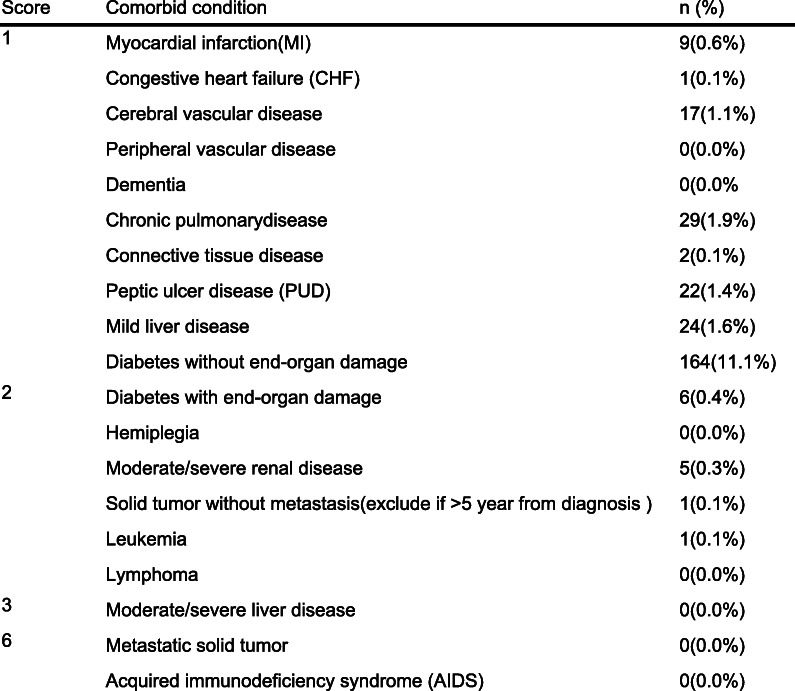
Age-Adjusted Charlson Comorbidity Index (*N* = 1476)

Age adjustment: for each decade after 40 years, add 1 point to total score (i.e. 1 point forage group 50–59 years, 2 points for age group 60–69, etc.)

### Markers of systemic inflammation

Hematology and laboratory examinations were performed 1 week before surgery. Parameters including neutrophil count, lymphocyte count, platelet count, and albumin (Alb) levels were collected. NLR is defined as neutrophil count divided by lymphocyte count. PLR is defined as platelet count divided by lymphocyte count. LMR is defined as lymphocyte count divided by monocyte count. According to previous literature, cut-off values of NLR, PLR and LMR were defined as 4.0, 161.3 and 3.4 [21], respectively. Only 1017 patients with detailed preoperative registration of LMR, NLR and PLR in the whole group (1476 cases) were included in analysis.

### Follow-up evaluation

Patients were followed up postoperatively using physical examination and laboratory tests, including tests for tumor markers (e.g., carcinoembryonic antigen [CEA] and CA19–9) every 3 months for the first 2 years, every 6 months for the next 3 years, and then annually. OS was defined as the time from surgery to the last follow-up or the date of death. Cancer-specific survival (CSS) refers to the time from surgery to the data of death from GC. Recurrence was diagnosed as an imaging manifestation or a biopsy of a suspicious lesion. Recurrence patterns were classified as local (anastomotic or gastric remnant), lymph node, peritoneum or hematogenous and unclear [22, 23]. Tumors involving the ovaries (Krukenberg’s tumor) were considered peritoneal [21].

### Statistical analysis

The statistical analyses were performed using SPSS version 22.0 (SPSS Inc., Chicago, IL, USA). PMS was uesd to to reduce confounding bias [24–27]. A logistic regression model was chose for calculating the propensity scores and the following covariates: tumor size, tumor location and tumor stage. The 1:1 matching process was peformed by using the nearest neighbor matching method, under a 0.2 caliper [28]. The optimal cutoff point of the ACCI was obtained through X-tile (Version 3.6.1, Yale University) [29, 30]. Categorical data was presented as proportions and analyzed with the Chi-square test or Fisher’s exact test. Survival rate was calculated with the Kaplan-Meier method, and the differences were compared by log-rank tests. The Cox proportional hazards model was used to assess differences in survival. Variables with a *p* value of < 0.05 on univariate analysis were then included in a multivariate Cox stepwise regression analysis. Data of whole cohort (1476 cases) were randomly divided into modeling group (70%) and validation group (30%) [31, 32]. A nomogram was established based on the Cox proportional hazards regression model for calculating risk score of independent prognosis factors to further evaluate predictive value of ACCI by R software version 3.4.3 (http://www.r-project.org/) with the survival, rms, and survivalROC package [33, 34]. The differences of predictive ability on survival were compared by the C-index.

## Results

### The distribution and cut-off of the ACCI

The ACCI in the whole cohort ranged from 0 to 8, with a median of 2.00. The optimal cutoff value of the ACCI determined by the X-tile program, based on OS, was 3.00 (X^2^ = 37.30, *P* < 0.05, Additional file 4: Figure S1 A and B). Thus, patients were categorized into two groups (ACCI category): a low ACCI group (ACCI = 0–2; 1047 cases, 70.9%), and a high ACCI group (ACCI = 3–8; 429 cases, 29.1%). As is shown in Additional file 1: Table S1, low ACCI group included patients who were younger than 70 years old without comorbidity or with low CCI (CCI < 3). Those who were older than 70 years old were all included in high ACCI group. With the increase in age, the proportion of patients with comorbidities is also increased.

### Clinicopathologic characteristics of the study population

The clinicopathologic characteristics of the whole cohort (1476 cases) and patients after PSM (858 cases) are shown in Table 2. High ACCI group and low ACCI group had significant differences in preoperative abdominal surgery history, ASA grade, tumor size, tumor location, pathological stages and histologic type (all *P* < 0.05), as well as in age (*P* < 0.001) and comorbidity (*P* < 0.001). After PSM, the difference in tumor size, tumor location and tumor stage between the two groups was not statistically significant. Before and after PSM, the incidence of postoperative complications in the high ACCI group was significantly higher than that in low ACCI group (*P* < 0.05). In addition, we also found significant differences in the distribution of NLR, LMR and PLR between the two groups, which may indicate the correlation between ACCI and systemic inflammation (*P* < 0.05, Additional file 2: Table S2).

**Table 2:**
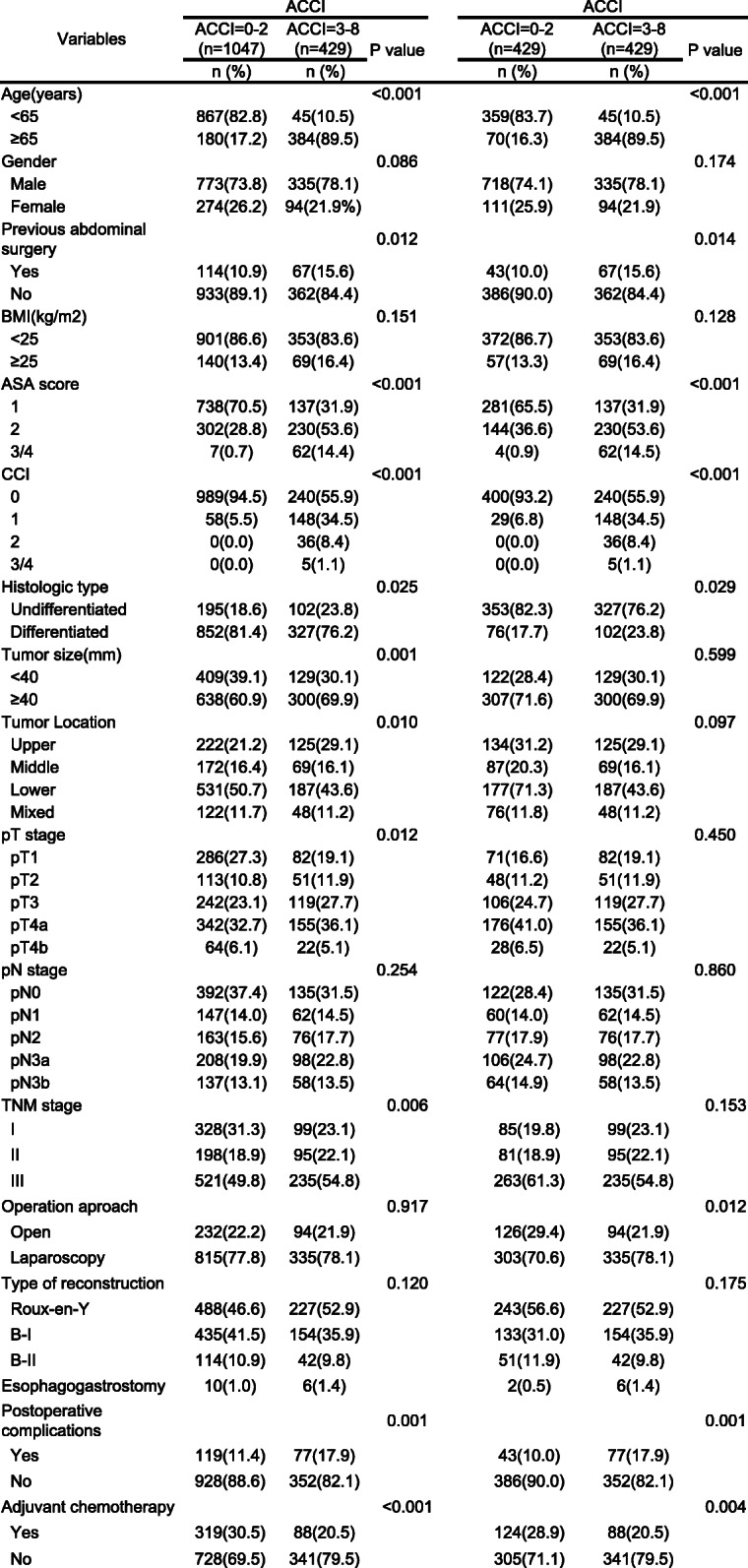
Clinicopathologic characteristicsbefore and after matching

*BMI* body mass index, *ASA* American Society of Anesthesiologists

### Influence of ACCI level on the prognosis of patients

**Fig. 1:**
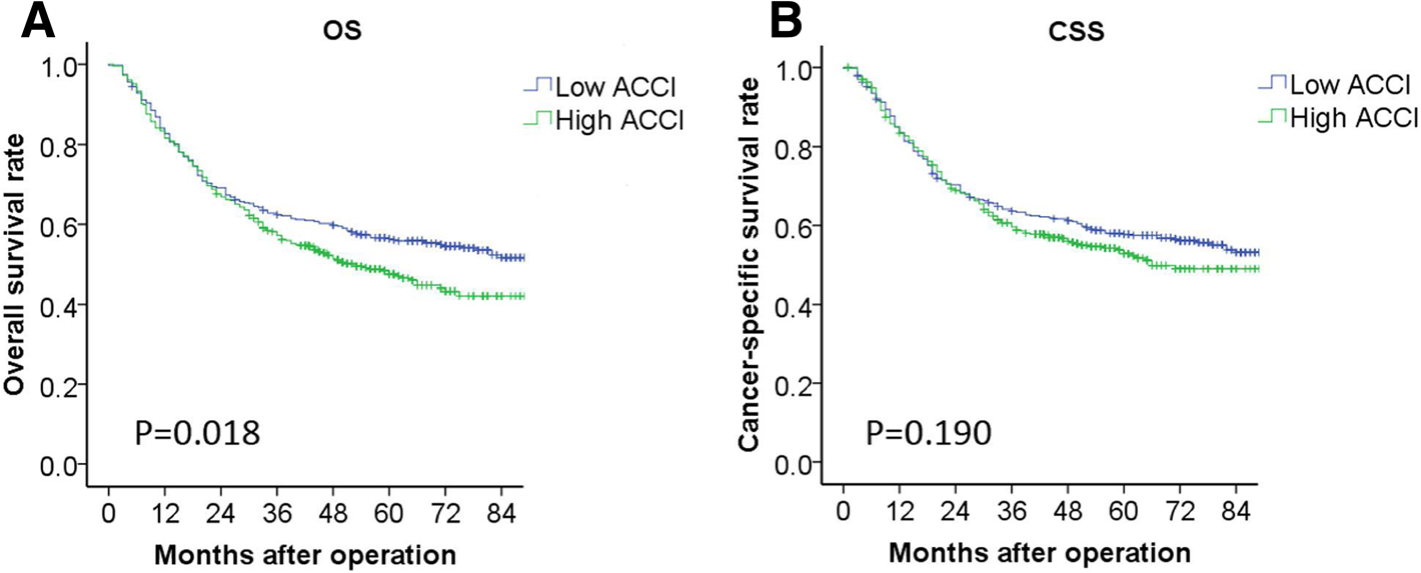
Kaplan-Meier overall survival (**a**) and cancer specific survival (**b**) for the Age-adjusted Charlson Comorbidity Index

**Fig. 2:**
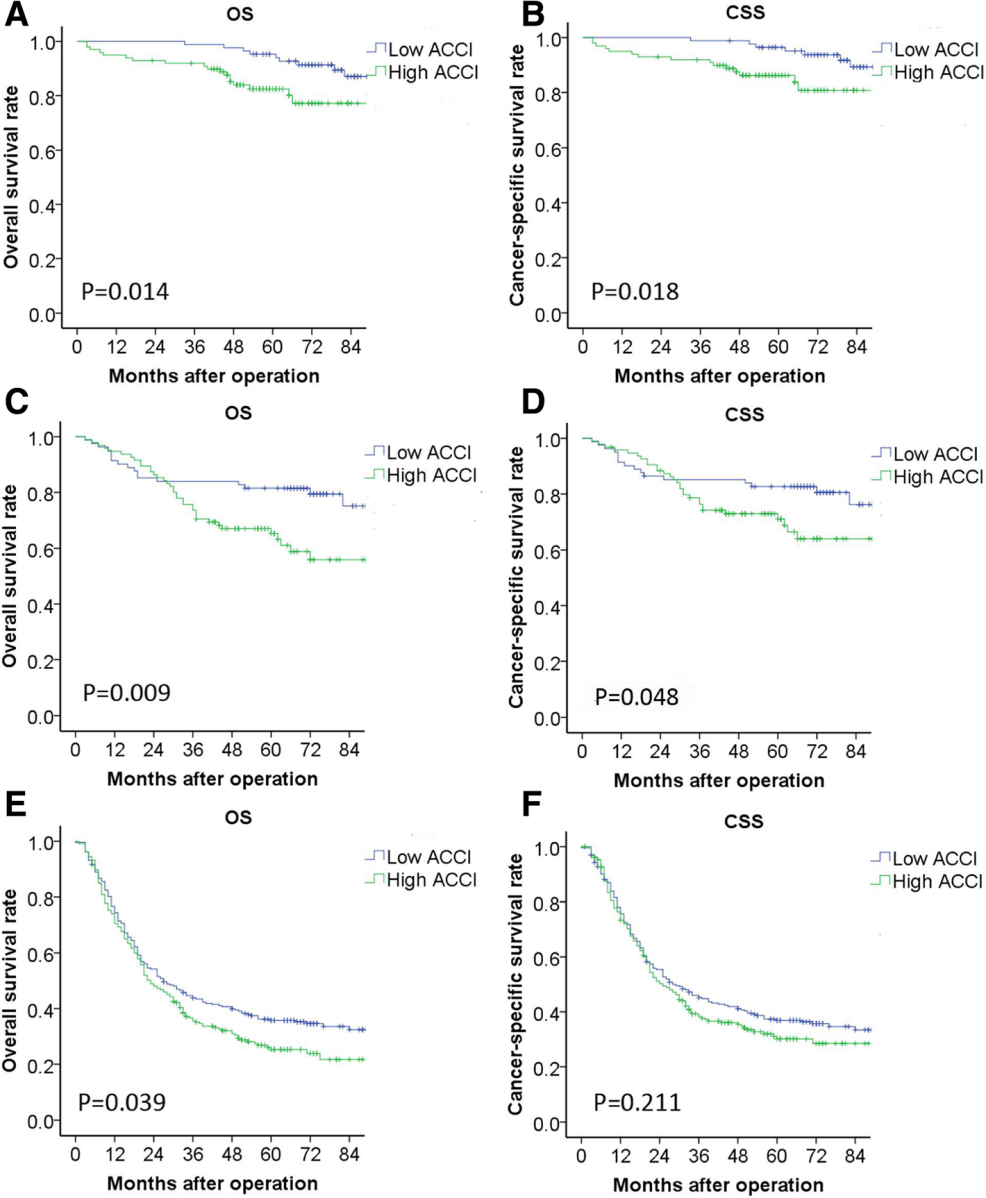
Kaplan-Meier (KM) curves comparing the OS of patients with low ACCI and high ACCI. (**a**) American Joint Committee on Cancer, 8th edition (AJCC) stage I, (**b**) AJCC stage II, and (**c**) AJCC stage III. (**d**-**f**) KM curves comparing the CSS of patients with low ACCI and high ACCI at: (**d**) AJCC stage I, (**e**) AJCC stage II, and (**f**) AJCC stage III

The median follow-up time of 858 matched patients was 48 months. There were significant differences in OS between high ACCI group and low ACCI group respectively (1-year OS: 82.7% vs 81.6%, 3-year OS: 62.4% vs 57.2%, 5-year OS: 56.4% vs 47.5%, all *P* < 0.05, Fig. 1). Although the difference of CSS between low and high ACCI groups were not reached statistic significant, the CSS curve in low ACCI group was still better than high ACCI group. Further stratified analysis showed that ACCI had a significant impact on the OS and CSS in patients with stage I and II. But in patients with stage III, significant difference only observed in OS between different groups, but not in CSS (Fig. 2). The types of tumor recurrence and metastasis observed during the follow-up period were shown in Additional file 3: Table S3. There were no statistically significant differences in the recurrence rate and the patterns of recurrence between patients of two groups with high ACCI and low ACCI (*P* > 0.05). However, high ACCI group still showed a trend of higher recurrence rate.

### Analysis of prognostic factors for OS and CSS

On univariate analysis, factors associated with 5-year OS included the ACCI, preoperative BMI, operative approach, tumor location, tumor size, TNM stage, histologic type and postoperative adjuvant chemotherapy (all *P* < 0.05, Table 3). Further multivariate analysis suggested that ACCI, tumor location, and TNM stage remained independent risk factors for OS (Table 4, all *P* < 0.05). While ACCI was not an independent prognostic factor for CSS (*P* > 0.05, Table 4).

**Table 3:**
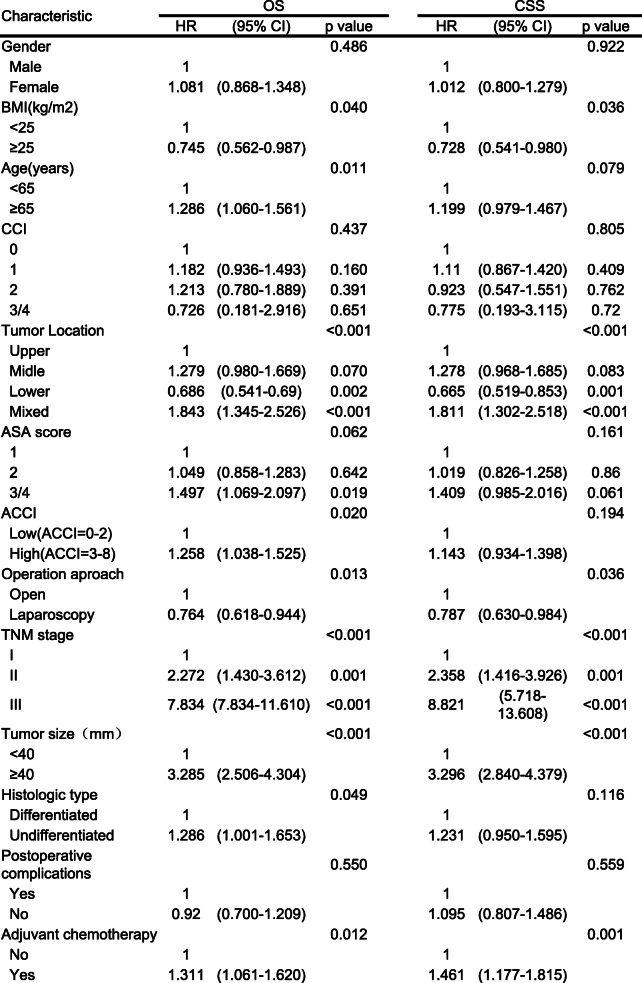
Univariate analysis of prognostic factors for OS and CSS

**Table 4:**
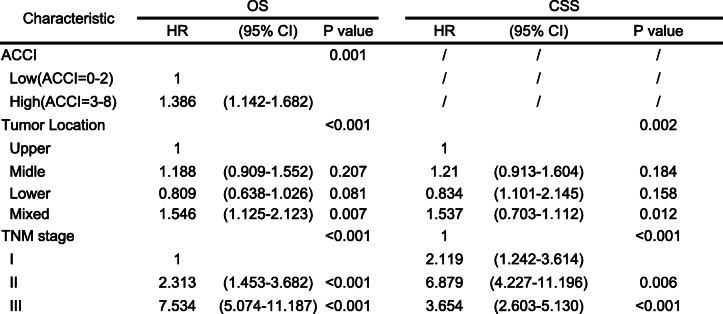
Multivariate analysis of prognostic factors for OS and CSS

### Predictive value of the ACCI on long-term prognosis

**Fig. 3:**
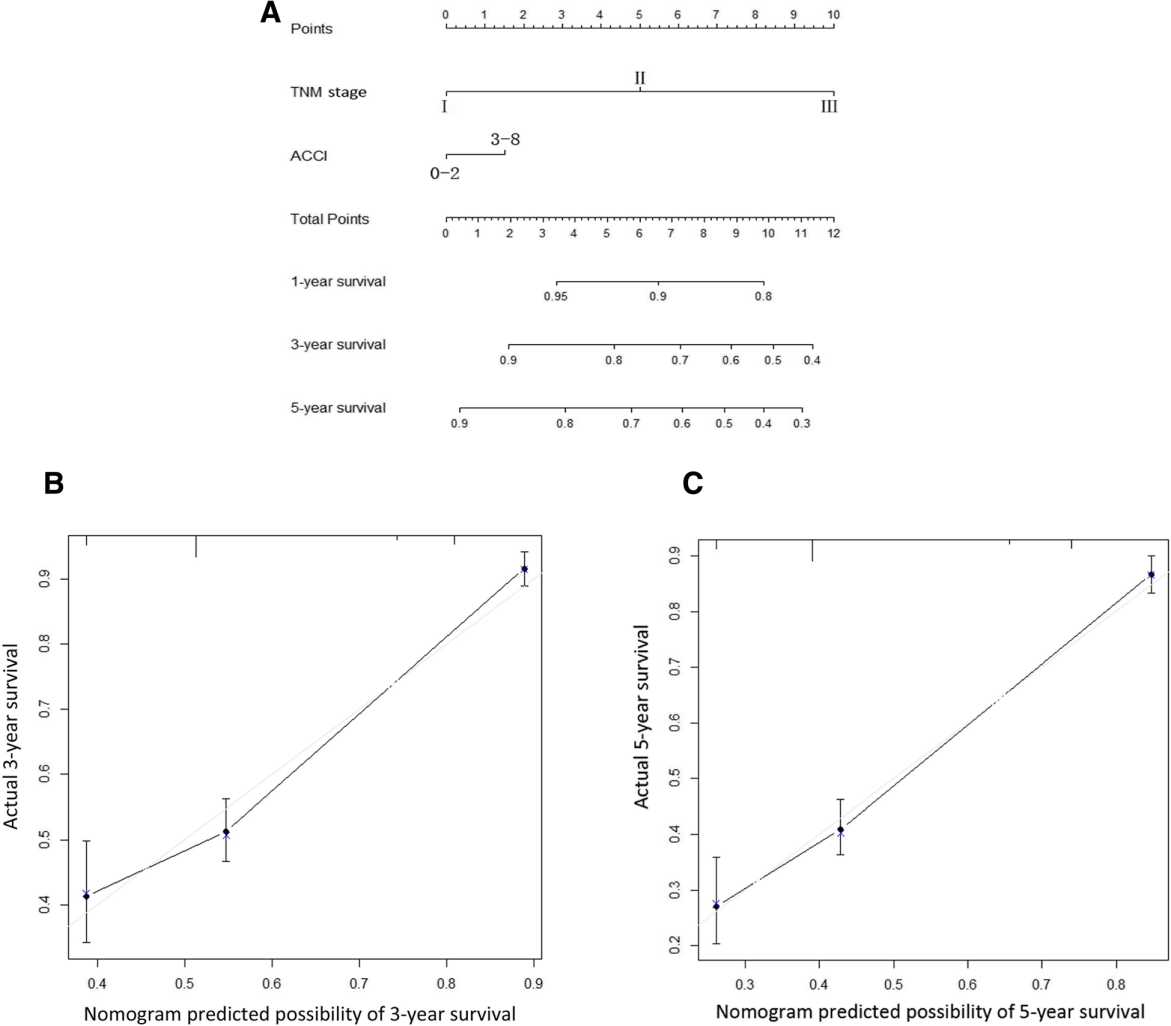
Nomogram plots and calibration curves based on ACCI and AJCC stage (**a**, **b**, **c**)

In order to further assess the predictive value of the ACCI on survival after radical gastrectomy in patients with GC, we combined the ACCI and TNM stage, developing a new predictive system (TNMA). A nomogram was developed to predict the 5-year OS, which was based on the independent prognostic factors identified by multivariate analyses (Fig. 3a). And statistically significant difference in C-index between TNMA and TNM staging system was observed (0.720 vs 0.708, *P* = 0.022). In validation group, C-index of TNMA for OS prediction was also significantly higher than that in the TNM staging system (0.777 vs 0.742, *P* < 0.001). It suggested that the new TNMA prediction system could better predict the long-term survival of GC patients than TNM stage.

## Discussion

With the aging of the population, the proportion of elderly patients with gastric cancer is gradually increasing. Meanwhile, the functional reserve of the human body tends to decline with age, which makes the incidence of complications increase [4]. Studies have shown that the screening strategy, treatment regimen and prognosis of patients with gastrointestinal tumors could be greatly affect by comorbidities [17]. The CCI was originally developed to predict the prognosis of patients with comorbidity in longitudinal studies [13]. It has been used in multiple studies to stratify the prognosis of patients and allow a more individualized treatment strategy. The ACCI is a simple scoring system further combining the two related factors of age and comorbidity. The previous study was mainly used to predict the occurrence of perioperative complications. Recently, studies has reported that ACCI also has predictive value on the long-term prognosis of patients with many malignant tumors including ovarian cancer, prostate cancer and lung cance [12, 13, 35]. However, the predictive value of ACCI on the long-term prognosis of patients with GC has not been reported. Therefore, in this large-scale study, we included patients with GC who underwent gastrectomy by a unified team to investigate the impact of ACCI on the long-term prognosis of GC patients.

In the previous manuscripts about the ACCI for the prediction of long-term prognosis of patients with malignant tumors, the selection of the optimal ACCI cut-off was quite different. Dias-Santos et al. obtained the cut-off point based on the ROC curve and quartile method [15], while Kahl et al. directly quoted similar studies in the literature for the cut-off [12]. In our study, the X-tile program and observed long-term survival of the study patients were used to obtain the ACCI cut-off value. This software, developed by Robert Camp et al. from Yale University in 2004, is a new method for obtaining the optimal cut-off. The cut-point selection is complicated by time-dependent assessment of the outcome in the X-tile program [30]. Compared with the ROC curve method for obtaining the optimal cutoff, which was merely based on the outcome, the use of the X-tile program is more appropriate. Thus, we obtained the best cut-off value of the ACCI at 3.00 and divided the whole cohort into a low-ACCI group (ACCI = 0–2) and high-ACCI group (ACCI = 3–8).

Since Virchow first established a link between cancer and inflammation in the nineteenth century, an increasing number of studies have shown that inflammatory markers is also plays an important role in tumor progression and metastasis [36]. It has been shown that preoperative inflammatory makers, including LMR, NLR and PLR, are closely related to the prognosis of various tumors [37–39]. Therefore, we included 1017 patients with detailed preoperative registration of LMR, NLR and PLR in the whole group for analysis, and found statistical differences in the distribution of inflammatory markers between low ACCI group and high ACCI group, which may indicate the correlation between ACCI and systemic inflammation.

Similar to most previous studies, the incidence of postoperative complications in our study was significantly higher in patients with a high ACCI [9, 12, 13], indicating that the ACCI had a significant effect on the short-term outcome of patients. In the prognostic analysis, the 5-year OS in high ACCI group were significantly worse than that in low ACCI group. Further stratified analysis showed that ACCI had a significant impact on the OS and CSS in patients with stage I and II. But in patients with stage III, significant difference only observed in OS between different groups. Although the CSS of low ACCI group is better than high ACCI group, the difference is not statistically significant. It also showed that there were no statistically significant difference in the recurrence rate and the patterns of recurrence between patients of two groups with high ACCI and low ACCI. However, high ACCI group still showed a trend of higher recurrence rate. This result may be affected to some extent due to not counting into the population with unclear site of recurrence. In the univariate analysis, ACCI and age were statistically significant prognostic factors for gastric cancer patients. On multivariate analysis, the ACCI was also an independent prognostic factor for gastric cancer patients, while age and CCI were not independent prognostic factors. This result demonstrated that ACCI, as a comprehensive indicator with age and comorbidity, provides a better prognostic assessment for patients. Although the effect of age and comorbidity should be assessed separately in a univariate, bivariate or multivariate analysis. The index is designed to be a simple, crude combined risk assessment for clinicians to use [10]. However, it is worth considering that, a stratified analysis showed that among patients without adjuvant chemotherapy, difference of 5-year OS between low ACCI group and high ACCI group was statistically significant. However, among patients received adjuvant chemotherapy, there was no statistically significant difference in survival between the two groups. These results might partly contribute to that the patients with a high degree of ACCI were more likely in poor physical condition than low ACCI patients. They may resist a more intensive treatment, which may affect the efficacy of treatment. This partly reflects the impact of ACCI on the treatment choices of patients after operation and their long-term prognosis. In addition, previous studies have shown that postoperative complications affect the long-term prognosis of patients [40, 41], while Liu et al. found that complications are unrelated to the long-term prognosis of gastric cancer patients [42], which is similar to our study. The relationship between postoperative complications and prognosis of gastric cancer patients is still controversial. Further analysis is needed on whether postoperative complications affect long-term prognosis.

An accurate staging system is essential for the prognosis of patients and the choice of treatment strategy options. The TNM staging system released by the AJCC is one of the most commonly used staging systems in the world. However, this system mainly focuses on the tumor condition, and personal factors are not included to make a more individualized evaluation. In this study, the combination of the ACCI and TNM stage (TNMA) was used to further evaluate the predictive value of the ACCI on prognosis in patients with GC. The results showed that C-index of TNMA on predicting OS of patients with GC was significantly higher than that in TNM staging system both in the modeling group and the validation group. Therefore, in adding the ACCI personal factors to the TNM staging system, the prediction of survival in patients with GC will be more accurate, and guidance will be enhanced for later treatment strategies. However, this study is a single-center study and lack of external validation, which should be validated by a multicenter prospective study.

## Conclusions

In conclusion, ACCI was an independent risk factor for the long-term prognosis of patients after radical gastrectomy. Moreover, ACCI could effectively improve the predictive efficacy of the TNM staging system for the prognosis of patients with GC. It provided a simple and effective tool for preoperative evaluation.

## Additional files

Additional file 1: **Table S1.** Distribution of ACCI score according to age and comorbidity. (PDF 78 kb)

Additional file 2: **Table S2.** Association of immune function and ACCI (*N* = 1017). (PDF 66 kb)

Additional file 3: **Table S3.** Distribution of site of recurrence between different ACCI groups. (PDF 66 kb)

Additional file 4: **Figure S1.** X-tile analysis of survival data reveals a continuous distribution based on the Age-Adjusted Charlson Comorbidity Index (ACCI) (A) X-tile plots for the ACCI constructed according to patients enrolled in this study. The plots show the X2 log-rank values with groups divided into 2 based on 1 cutoff points. The brightest pixel represents the maximum X2 log-rank value (37.298) generated by the cutoff value (3.00) as marked by the black spot. (B) The distribution of the number of patients related to ACCI. The ACCI ranged from 0.00 to 8.00 with a median of 2.00. (C) Survival curve of patients according to the ACCI. (PDF 196 kb)

## Abbreviations

ACCI: Age-Adjusted Charlson Comorbidity Index
AJCC: American Joint Committee on Cancer
Alb: albumin
ASA: American Society of Anesthesiologists
BMI: body mass index
CCI: Charlson Comorbidity Index
CEA: carcinoembryonic antigen
C-index: the Harrell’s C-statistics
CSS: cancer-specific survival
GC: gastric cancer
LMR: lymphocyte count divided by monocyte count
NLR: neutrophil count divided by lymphocyte count
OS: overall survival
PLR: platelet count divided by lymphocyte count
PSM: propensity score matching
TNMA: a predictive system combining the ACCI and TNM stage
