# Supplementary material for: Age-adjusted Charlson Comorbidity Index (ACCI) is a significant factor for predicting survival after radical gastrectomy in patients with gastric cancer
Source: BMC Surg. 2019 May 27;19:53. doi: 10.1186/s12893-019-0513-9 (PMC6537159; doi:10.1186/s12893-019-0513-9)
Supplement: Supplementary file 1 — Table S1. Distribution of ACCI score according to age and comorbidity. (PDF 78 kb) [file 12893_2019_513_MOESM1_ESM.pdf]

Supplemental Table 1. Distribution of ACCI score according to age and comorbidity.

Age	CCI= 0	CCI=1	CCI=2	CCI=3	CCI=4
≤49	0	1	2	3	4
	(220,95.7%)	(10,4.3%)	(0,0.0%)	(0,0.0%)	(0,0.0%)
50-59	1	2	3	4	5
	(363,86.8%)	(48,11.5%)	(6,1.4%)	(1,0.2%)	(0,0.0%)
60-69	2	3	4	5	6
	(406,83.2%)	(70,14.3%)	(11,2.3%)	(1,0.2%)	(0，0.0%)
70-79	3	4	5	6	7
	(200,70.6%)	(69，24.4%)	(13，4.6%)	(1，0.3%)	(0，0.0%)
≥80	4	5	6	7	8
	(40，70.2%)	(9，15.8%)	(6，10.5%)	(1，1.8%)	(1，1.8%)
