# Supplementary material for: Age-adjusted Charlson Comorbidity Index (ACCI) is a significant factor for predicting survival after radical gastrectomy in patients with gastric cancer
Source: BMC Surg. 2019 May 27;19:53. doi: 10.1186/s12893-019-0513-9 (PMC6537159; doi:10.1186/s12893-019-0513-9)
Supplement: Supplementary file 2 — Table S2. Association of immune function and ACCI (N = 1017). (PDF 66 kb) [file 12893_2019_513_MOESM2_ESM.pdf]

Supplemental Table 2. Association of immune function and ACCI(N=1017)

Variables	ACCI		P value
	ACCI=0-2(n=734)	ACCI=3-8(n=283)	
	n (%)	n (%)	
LMR			<0.001
＜3.4	519(70.7%)	161(56.9%)	
≥3.4	215(29.3%)	122(43.1%)	
NLR			0.015
＜4.0	662（90.2%）	240（84.8%）	
≥4.0	72（9.8%）	43（15.2%）	
PLR			<0.001
＜161.3	192(26.2%)	120(42.4%)	
≥161.3	542(73.8%)	163(57.6%)	
