# Supplementary material for: Age-adjusted Charlson Comorbidity Index (ACCI) is a significant factor for predicting survival after radical gastrectomy in patients with gastric cancer
Source: BMC Surg. 2019 May 27;19:53. doi: 10.1186/s12893-019-0513-9 (PMC6537159; doi:10.1186/s12893-019-0513-9)
Supplement: Supplementary file 3 — Table S3. Distribution of site of recurrence between different ACCI groups. (PDF 66 kb) [file 12893_2019_513_MOESM3_ESM.pdf]

Supplemental Table 3. Patterns of recurrence between different ACCI groups

patterns of recurrence	ACCI		P value
	ACCI=0-2(n=429)	ACCI=3-8(n=429)	
	n (%)	n (%)	
Local			0.929
yes	43(28.3%)	18(27.7%)	
no	109(71.7%)	47(72.3%)	
Lymphonodus			0.757
yes	23(5.4%)	21(4.9%)	
no	406(94.6%)	408(95.1%)	
hematogenous			0.213
yes	59(13.8%)	47(11.0%)	
no	370(86.2%)	382(89.0%)	
Peritoneum			0.623
yes	21（4.9%）	18（4.2%）	
no	408（95.1%）	411（95.8%）	
Unclear			0.090
yes	104（24.2%）	126（29.4%）	
no	325（75.8%）	303（70.6%）	
