# Supplementary figures and images for: Age-adjusted Charlson Comorbidity Index (ACCI) is a significant factor for predicting survival after radical gastrectomy in patients with gastric cancer
Source: BMC Surg. 2019 May 27;19:53. doi: 10.1186/s12893-019-0513-9 (PMC6537159; doi:10.1186/s12893-019-0513-9)

Supplemental Fig. 1

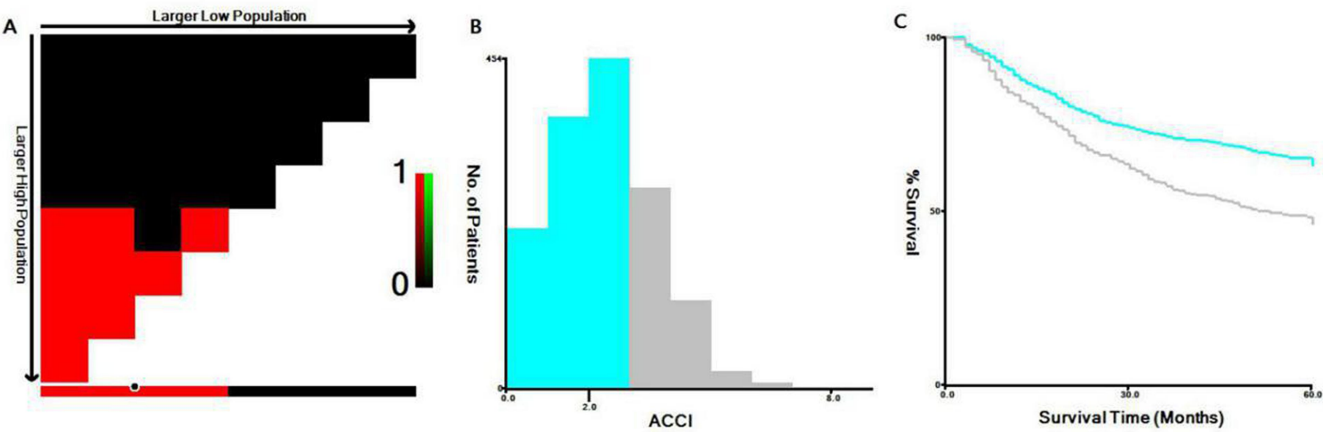

Supplement: Supplementary file 4 — Figure S1. X-tile analysis of survival data reveals a continuous distribution based on the Age-Adjusted Charlson Comorbidity Index (ACCI) (A) X-tile plots for the ACCI constructed according to patients enrolled in this study. The plots show the X2 log-rank values with groups divided into 2 based on 1 cutoff points. The brightest pixel represents the maximum X2 log-rank value (37.298) generated by the cutoff value (3.00) as marked by the black spot. (B) The distribution of the number of patients related to ACCI. The ACCI ranged from 0.00 to 8.00 with a median of 2.00. (C) Survival curve of patients according to the ACCI. (PDF 196 kb) [file 12893_2019_513_MOESM4_ESM.pdf]
